# Truly “Rational” Polytherapy: Maximizing Efficacy and Minimizing Drug Interactions, Drug Load, and Adverse Effects

**DOI:** 10.2174/157015909788848929

**Published:** 2009-06

**Authors:** Erik K St. Louis

**Affiliations:** Mayo Clinic, Rochester, Minnesota, USA

**Keywords:** Epilepsy, antiepileptic drugs, polytherapy, drug load, drug interactions.

## Abstract

While several newer AEDs have study data that support monotherapy usage, most possess FDA indications for adjunctive treatment of partial onset seizures, leading to their initial (and often persistent) clinical use as adjunctive polytherapy for patients with refractory epilepsy. This review considers a practical approach to the appropriate role for polytherapy in epilepsy, presents the evidence for AED polytherapy, reviews the mythic but practically reasonable concept of “rational polytherapy,” and concludes with practical strategies for avoiding and employing polytherapy in clinical practice. The appropriate indications for AED polytherapy include transitional polytherapy during titration of a new adjunctive AED toward monotherapy or long-term maintenance AED polytherapy in medically refractory epilepsy.

## INTRODUCTION

This is an exciting time to treat patients with epilepsy. The advent of an impressive and ever-evolving armamentarium of newer (second- and third-generation) antiepileptic agents (AEDs) offers considerable advantages in safety and tolerability over older, first generation AEDs. Since 1994, eleven AEDs (felbamate, gabapentin, lacosamide, lamotrigine, levetiracetam, tiagabine, topiramate, oxcarbazepine, pregabalin, rufinamide, and zonisamide) have been approved by the Food and Drug Administration (FDA) based on pivotal, adjunctive therapy trial designs. While several second-generation AEDs have study data that support monotherapy usage, most possess FDA indications for adjunctive treatment of partial onset seizures, leading to their initial (and often persistent) clinical use in polytherapy. There is not yet ample evidence to commend “third generation” AEDs (lacosamide and rufinamide) for routine use as monotherapy in clinical practice, so it is expected that the major use of these two newer AEDs will be as adjunctive polytherapy for patients with refractory epilepsy.

This review considers a practical approach to the appropriate role for polytherapy in epilepsy, presenting the evidence for AED polytherapy, reviewing the mythic but practically reasonable concept of “rational polytherapy,” and concluding with practical strategies for avoiding and employing polytherapy in clinical practice. The appropriate indications for AED polytherapy include transitional polytherapy during titration of a new adjunctive AED toward monotherapy or long-term maintenance AED polytherapy in medically refractory epilepsy.

## EVIDENCE SUPPORTING POLYTHERAPY

Initial AED monotherapy is effective in rendering approximately 60% of epilepsy patients seizure free [34]. The remaining patients are considered medically refractory and are candidates for polytherapy, surgery, or vagus nerve stimulation (VNS). Based on this information, at least 14% of epilepsy patients will be polytherapy candidates.

The American Academy of Neurology/American Epilepsy Society (AAN/AES) Practice Guidelines for the treatment of refractory epilepsy supports the use of second-generation AEDs for adjunctive treatment of refractory partial-onset seizures in adults [24]. There is ample evidence to conclude that nearly all currently marketed AEDs are effective for the adjunctive treatment of refractory partial seizures (except ethosuximide, which is only effective for treatment of generalized absence seizures, and in particular lacks efficacy for treatment of partial-onset seizures). However, an ideal combination of AEDs has not been identified. While meta-analyses results have indicated that lamotrigine, oxcarbazepine, and topiramate demonstrate overlapping efficacy; direct comparison between individual trials is not possible due to important baseline differences in patient demographics, seizure frequency, disease severity, and inclusion/exclusion criteria [11,24,46].

Other than the recent randomized, blinded, controlled trials of the second- and third-generation AEDs, most polytherapy studies were retrospective case series and uncontrolled trials. Historically, comparative polytherapy trials have found that between 15% to 35% of patients with partial seizures become seizure-free, and an additional 12% to 29% of patients have a >50% seizure reduction with polytherapy [12,15,26,31,41,54,73,79]. The VA I Cooperative Trial, a prospective, randomized trial, found that 40% of patients failing phenytoin or carbamazepine monotherapy respond to polytherapy, with 11% of these patients becoming seizure-free [48].

## WHAT IS RATIONAL POLYTHERAPY?

The concept of “rational polytherapy” has held that AED combinations with differing mechanisms of action are more effective than polytherapy with similar mechanisms of action [40,60,66]. An early attempt at rational polytherapy was the 19^th^ century combination therapy nervine (a mixture of bromide, arsenic, and picrotoxin); later, co-therapy with phenytoin and phenobarbital was commonly used for new-onset epilepsy during much of the 20^th^ century [10,30,74]. Rational polytherapy is a logical concept, since the pathophysiology of epilepsy is believed to be consequent to two opposing types of neural imbalances. Patients may either exhibit excessive neuronal excitation mediated by pro-glutamatergic influences (the principle excitatory neurotransmitter) or a failure of inhibition due to decreased GABA-ergic activity (the main inhibitory neurotransmitter). AED combinations that target over exuberant excitation and insufficient inhibition would be expected to be more efficacious. Polytherapy can produce additive, antagonistic, or synergistic efficacy and toxicity [14]. AEDs with similar mechanisms of action would be expected to produce merely additive efficacy, while AEDs with differing mechanisms of action would be expected to be synergistic. Combining AEDs with competitive hepatic enzymatic metabolism or protein binding may produce antagonism of one or both AEDs’ efficacy or lead to heightened toxicity. AEDs with similar toxicity profiles could produce additive or synergistic pharmacodynamic adverse effects. Animal studies utilizing rational polytherapy have confirmed that both synergistic efficacy [47] and unexpected toxicity [64] may result with varying AED combinations.

Several pre-clinical experiments suggest synergistic efficacy between specific combinations of AEDs, including: phenytoin and phenobarbital; [5] topiramate with carbamazepine, felbamate, lamotrigine, phenobarbital, or tiagabine; [14] valproate with phenytoin, carbamazepine, or ethosuximide; [9,14,16] and AEDs with calcium channel inhibitors or N-methyl d-aspartate (NMDA)-type glutamate receptor antagonists [14]. Levetiracetam may also have synergism with several AEDs, particularly valproate [47]. Some AED interactions operate in an unpredictable antagonistic fashion; for example, ethosuximide and valproate exhibit an increased threshold for toxicity [64].

An example of rational polytherapy would be levetiracetam, which modulates pre-synaptic neurotransmitter release *via* synaptic vesicle protein 2A (SV2A) binding, [42-43] plus carbamazepine, a sodium channel ionophore complex inactivator which limits neuronal burst firing and seizure discharge propagation. A review of both polytherapy retrospective case series and clinical trials found low-grade evidence favoring the combination of sodium channel blocking AEDS and those with γ-aminobutyric acid (GABA) activity [17]. On the other hand, combining two GABA mimetic drugs or alpha-amino-3-hydroxy-5-methyl-4-isoxazolepropionic acid (AMPA) and N-methyl-D-aspartic acid (NMDA) antagonists may enhance efficacy but reduce tolerability [17]. Also, despite the theoretical advantages of utilizing rational polytherapy, in most instances, AED combinations have not demonstrated an improved therapeutic index (efficacy/toxicity ratio) over monotherapy [6]. While the rational polytherapy approach is certainly sensible and commonly employed in clinical practice, there is no evidence from clinical trials to support and justify its use. One previous clinical trial has suggested the potential for drug synergy and practical existence of “rational polytherapy” in a clinical population [7]. The study enrolled patients receiving one of four older AEDs (carbamazepine, phenobarbital, phenytoin, or valproate) in monotherapy. Subjects then received adjunctive lamotrigine, and patients responding with a 50% or greater seizure reduction were subsequently converted to monotherapy with lamotrigine. Efficacy analysis demonstrated that combination polytherapy with valproate and lamotrigine was more effective than other combinations (valproate-lamotrigine subgroup 64% responders, carbamazepine-lamotrigine 41% responders, phenytoin 38% responders), suggesting potential synergy between. However, the study was not designed to explore synergy between any of the drugs, and drug interaction alone (i.e., higher lamotrigine plasma concentrations mediated by concurrent valproate administration) was an alternative, and more parsimonious explanation, for higher efficacy with a combined valproate-lamotrigine regimen [7].

Table**1** lists the proposed pharmacological targets of commonly used AEDs and serves as a reference for choosing combinations of AEDs with complementary mechanisms of action with respect to the practical principle of “rational polytherapy” (despite a current lack of evidence basis for this approach).

## APPROPRIATE POLYTHERAPY: A PRACTICAL APPROACH

There are no randomized trials to suggest superior efficacy of polytherapy over monotherapy. Since polytherapy often affords only modest improvement in efficacy, but significant increases in adverse effects, it is sensible to first think of strategies for avoiding polytherapy. Most patients should receive two sequential trials of AED monotherapy, utilizing AEDs with differing mechanisms of action, prior to attempting chronic polytherapy.

Failure to produce seizure control after even one well-tolerated, optimally administered monotherapy AED trial is an ominous prognostic feature, suggestive of medically refractory epilepsy [35]. After one or two failed trials of monotherapy, all patients deserve additional investigations to ensure that the correct epilepsy syndrome has been diagnosed, and to exclude the possibility of an alternative diagnosis of a medical or psychiatric mimicker of epilepsy. In particular, the possibility of psychogenic non-epileptic spells (pseudoseizures), seen in 20-50% of epilepsy monitoring practices, needs to be considered, as many such patients present after an erroneous diagnosis of epilepsy for several years and have been treated with polytherapy, subjecting these patients to unnecessary risk and adverse effects [33].

**Fig. (1):**
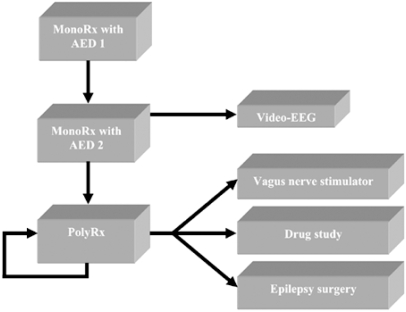
**Therapeutic Triage in Epilepsy Care.** Newly diagnosed epilepsy patients are most appropriately treated with antiepileptic drug monotherapy. Following failure of a second monotherapy, clinicians should implement courses of sequential chronic maintenance polytherapy and intensive evaluation to ensure correct epilepsy syndrome diagnosis and exclusion of nonepileptic spells. Pre-surgical evaluation with seizure-protocol magnetic resonance imaging (MRI) of the brain and ictal video-EEG recording of the patient’s habitual clinical spells should also be strongly considered to evaluate the patient’s potential to benefit from non-pharmacologic treatment options such as epilepsy surgery and VNS.

With refractory partial epilepsy, triage to non-pharmacological treatments including epilepsy surgery and vagus nerve stimulation (VNS) should be strongly considered, since these treatments may afford opportunities to reduce or eliminate polytherapy, improve quality of life, and reduce cost of treatment [3,4,32,36]. Polytherapy in women of childbearing potential is a particular concern given the heightened risk of teratogenesis [13,57,59]. A suggested algorithm for initial epilepsy treatment, showing the steps following unsuccessful monotherapy toward either pursuing polytherapy or avoiding it by evaluation and pursuit of appropriate non-pharmacologic therapies, is illustrated in (Fig.**1**).

There is no evidence to support polytherapy in new-onset epilepsy. Polytherapy is indicated for medically refractory epilepsy, and, even then, polytherapy may have two different goals and courses is further outlined and discussed in Article 3 (“Transitional Polytherapy” by Garnett, *et al*., Fig. **1**, pg. 85 (or whatever the journal page # would be for the run). AED management may involve transitional polytherapy (conversion to a second-line monotherapy) or chronic maintenance polytherapy, possibly requiring additional AED sequencing to optimize seizure control and minimize adverse effects. Tenets of successful polytherapy include selecting co-therapies that lack drug-drug interactions, have a limited potential for amplification of adverse effects, and minimize total drug load to achieve desired seizure control. A possible algorithmic approach to initiating or terminating polytherapy is outlined in Fig. (**1**) on page 85 of the article "Transitional Polytheraphy: Tricks of the Trade for Monotherapy to Monotherapy AED Conversions" by Garnett, *et al.* in this issue. Examples of some potentially desirable AED combinations (that illustrate a “rational polytherapy” approach) and undesirable combinations (that increase the likelihood of untoward drug-drug interactions) are shown in Table**2**.

## BEFORE INITIATING POLYTHERAPY

When utilizing polytherapy, the clinician must be knowledgeable about the potential for pharmacokinetic and pharmacodynamic interactions, which influence the risk of developing adverse effects. While an exhaustive review of drug interactions is beyond the scope of this article, a few illustrative scenarios will suffice to make this important point. In general, the main pharmacokinetic interactions to consider in AED polytherapy are potential cytochrome P450 (CYP) metabolism competition and a high percentage of protein binding that results in drug displacement. Co-administration of the enzyme-inducing AEDs (EIAEDs) (ie, phenobarbital, phenytoin, or carbamazepine) with inducible AEDs (such as lamotrigine, oxcarbazepine, tiagabine, topiramate, or zonisamide) hastens the metabolism of the latter, reducing drug concentrations and efficacy. Conversely, when valproate, an inhibitor of lamotrigine glucuronidation and clearance is given with lamotrigine, there is a greater chance of serious rash than when lamotrigine is given with EIAEDs [1,53]. Two recent studies of vulnerable institutionalized patients well illustrate the complex pharmacokinetic issues that can arise in polytherapy. In these studies of elderly nursing home and multiply handicapped patients, common use of undesirable pharmacokinetic AED combinations was found, especially phenytoin/phenobarbital polytherapy [27,45]. The interactions between these two AEDs are bidirectional, complex, and variable, often leading to unpredictable increases or decreases in drug concentrations. In an institutionalized patient population with common comorbid hypoalbuminemia, free phenytoin levels enable appropriate co-therapy adjustments and thereby avoid toxicity. In these and all patients, the goal should be to minimize the likelihood of complex drug interactions. Rather than rote memorization of a long list of potential drugs interacting with one another, it is more important and practical to have a working understanding of mechanisms for each drugs to interact with others that may allow one to predict or anticipate interactions; details on specific likely interactions can always be gleaned (and when in doubt, should be actively sought by practitioners) from comprehensive references such as the Physician’s Desk Reference (PDR), or online with *Micromedix*. Table **3** includes mechanisms for common AED interactions that are crucial to understand and be familiar with for use of the AEDs in clinical practice. For further references on these important considerations involved with initiating and maintaining polytherapy, the reader is referred to two recently published extensive reviews of AED drug interactions [22,58].

Pharmacodynamic adverse effects, such as dose-related neurotoxic and cognitive side effects, are especially difficult to avoid when using polytherapy [20]. Cognitive impairment is commonly seen with polytherapy and is often subtle and difficult to identify without specifically questioning the patient. While standard office assessment of cognition often shows minimal impact, detailed neuropsychological and electrophysiological measures may show impairments in attention, concentration, executive function, and memory in patients receiving AED therapy [50-52,62,70,77,78]. Some adverse effects such as sedation, cognitive impairments, gait disturbance, and hair changes are consistently underreported unless patients are specifically questioned about the presence or absence of these symptoms. Routine use of adverse event screening instruments during office visits aids in the identification of patient adverse effects that limit quality of life, especially for patients receiving polytherapy [25]. Readers are referred to the next article in this issue (“Minimizing Adverse Effects in Epilepsy Care”) for discussion and references on monitoring and reducing adverse effects of AEDs.

Some AEDs have a greater tendency to cause pharmacodynamic adverse effects when used in polytherapy; for example, there is an increase in the incidence of adverse effects if topiramate is utilized as adjunctive therapy than when it is administered as monotherapy [55,56,61,63]. Thus, before initiating polytherapy in a patient with epilepsy, the clinician should design a patient specific AED regimen that minimizes adverse events and drug interactions (by taking into account AED specific pharmacokinetics and pharmacodynamics), while maximizing efficacy, and continuously monitor that patient for signs of toxicity.

## HOW TO INITIATE POLYTHERAPY

A commonly employed method of introducing an adjunctive drug is to hold the current AED at a constant dose, then gradually titrate the new AED to the target dose [72]. Rapid dose escalation has been associated with AED therapy discontinuation, so most AEDs should be initiated at a low dose, and increased slowly to maximize patient tolerability and avoid dose related side effects (eg, drowsiness, dizziness, ataxia, or visual problems). If adverse effects emerge during the titration of the adjunctive AED, there are two possible approaches: 1) reduce the baseline AED to “make room” for continued titration of adjunctive therapy; dose-related adverse effects may be pharmacodynamically mediated by both AEDs, not solely due to the new AED (ie, flexible dose approach) [56] or 2) reduce the new AED; thereby accepting a lower target dose of this therapy (similar to the fixed dose approach utilized in most partial seizure adjunctive therapy clinical trials). Recently, a randomized, prospective, adjunctive topiramate trial addressed adverse effects emerging during adjunctive AED titration and assessed whether a flexible or fixed titration approach was most tolerable [56]. Patients were randomized to one of two treatment arms: 1) a “Flex Dose” titration group, in which investigators were permitted to reduce the baseline AED dose as needed to permit titration of adjunctive topiramate; and 2) a “Fixed Dose” titration group, which did not allow investigators to adjust the baseline AED dose. If the investigators needed to adjust baseline co-therapy, the patient was removed from randomized treatment. The primary study endpoint was the percentage of patients dropping-out of randomized treatment due to adverse effects. The Flex Dose group achieved higher target doses of adjunctive topiramate, while the Fixed Dose group had nearly twice as many subjects drop-out because of intolerable adverse effects. This data suggests that reduction of the baseline AED dosage is a superior approach for minimizing adverse effects that emerge during the titration of a new adjunctive therapy, enabling an adequate therapeutic trial of the new adjunctive AED. An adjunctive AED can be further increased as needed to achieve optimal therapeutic doses. In many instances, it is sensible to transition to monotherapy with the newly added AED, by discontinuing the baseline AED [2]. Some advocate initial lowering of the baseline AED prior to initiating a new adjunctive AED [66]. While this approach may improve the patient’s ability to tolerate the new adjunctive AED, the risk of breakthrough seizures is increased by such an approach, leading a recently convened expert consensus panel of epileptologists to instead recommend holding an initial baseline drug at a constant dose during titration of a new adjunctive AED until the target dose of that drug is reached, followed by taper of the primary baseline drug unless adverse effects occur, in which case tapering the primary drug can be tapered and withdrawn [72]. An approach of “criss-crossing” and existing primary the new adjunctive AED (i.e., titrating the new AED while concomitantly withdrawing an existing baseline AED) is probably best reserved for patients who are known to have a heightened sensitivity to AED adverse effects.

Similar decisions about drug titration need to be made when considering the addition of a third AED to a patient’s regimen, or when fourth or fifth adjunctive AEDs are contemplated. Use of more than two AEDs is generally discouraged due to an increased likelihood of pharmacokinetic and pharmacodynamic AED interactions with each additional AED. A recent, large, retrospective study suggested that two or three AEDs may effectively control seizures, but four or more AEDs were not beneficial [69]. Data from previous clinical trials suggests that between 20% to 50% of patients benefit from triple AED polytherapy by achieving a 50% or greater reduction of seizures [20,29,49]. Unfortunately, currently available data does not provide conclusive evidence on how to initiate the third AED. One reasonable option is to maintain the baseline AED regimens, titrate the third AED to a target dose, and then taper off the least effective AED. If an adverse effect develops during titration of the newest AED, immediately taper off the least effective AED to improve tolerability. Alternatively, if one of the AEDs in the regimen can be singled out as ineffective or poorly tolerated, consider titrating the new AED while simultaneously tapering the ineffective or intolerable AED.

## WHEN POLYTHERAPY BECOMES OVER-TREATMENT

Polytherapy has been identified as one means of over-treatment in epilepsy. Over-treatment may be defined as an excessive number or amount of AED(s) given, that results in a suboptimal risk-to-benefit balance [20,67]. Tapering of one or more AEDs can be successfully accomplished in many patients receiving chronic polytherapy, without substantial loss of seizure control [2,8]. There are many reasons to consider reducing polytherapy, including reducing the risk of serious adverse effects, minimizing drug interactions [60], and decreasing costs [4]. Uncontrolled seizures and polytherapy have been linked to decreased quality of life [75]. Paradoxically, over-treatment with AEDs can occasionally result in an increase in seizure activity, and reduction in polytherapy has been shown to lead to improved seizure control in approximately two-thirds of patients [8,20].

Considerable research has demonstrated that the most deleterious outcome associated with polytherapy is an increased risk of adverse effects (eg, sudden unexplained death in epilepsy patients (SUDEP), memory complaints, depression, and fatigue) due to pharmacodynamic dose-related neurotoxic effects, drug interactions, additive or synergistic drug related toxicities, and teratogenicity [1,13,57,59,67]. Polytherapy may be less tolerable than monotherapy because of a higher total drug load [16,18]. Most polytherapy studies have not controlled for total drug load [16,17,20]. Total drug load is the sum of the prescribed daily doses (PDD) of the AEDs that an individual patient is receiving. If the ratio of the PDD and the average effective or defined daily dose (DDD) of the AED exceeds 2, patients are more likely to exhibit neurological adverse effects [16,21,39]. DDD values assigned by the World Health Organization (WHO) for the AEDs are listed in Table **4** [16,81]. Whether using one or multiple AEDs, the PDD/DDD ratio should be below 2 to reduce the likelihood of neurological side effects; when using more than one AED, doses should generally be below or at the DDD for each individual drug to maximize tolerability for the patient.

Data comparing tolerability of monotherapy and polytherapy support the theory that equalizing total drug load of monotherapy to polytherapy leads to similar adverse effects with both approaches [19,39]. A cohort study of patients receiving either monotherapy or polytherapy in comparable drug loads showed no difference in perceived adverse effects [39]. A prospective, randomized study comparing carbamazepine alone to carbamazepine plus valproate, using comparable drug loads, found no difference in tolerability or efficacy between the two groups [18]. This study demonstrates that equalizing drug loads between monotherapy and polytherapy regimens may improve patient tolerability of polytherapy and provide a standardized method to compare the efficacy of monotherapy and polytherapy regimens. This study could not conclude whether monotherapy is as effective as polytherapy since it was powered to assess tolerability (primary endpoint), and was not designed to detect a difference in seizure control. The drug load concept can be applied to clinical practice; when the addition of another AED is required, the PDD/DDD ratio provides a guidepost for the total dose that a patient would be most likely to tolerate. Potential problems with the total drug load concept include the failure to predict non-linear efficacy or tolerability due to pharmacodynamic drug interactions, and the inability to account for various pharmacokinetic drug interactions, including protein binding displacement and cytochrome dependent induction or inhibition [16]. Further research regarding application and utility of the drug load concept in clinical practice is necessary. Also, since the drug load concept has not been frequently considered in AED polytherapy, an attractive and tantalizing prospect for future polytherapy research would be the design of future randomized controlled trials comparing the efficacy and tolerability of polytherapy with monotherapy in newly diagnosed and refractory epilepsies, controlling for equivalent drug loads between monotherapy and polytherapy groups.

## REDUCING UNNECESSARY POLYTHERAPY

Polytherapy continues to be common practice, especially in institutionalized epilepsy patients. Practitioners should regularly and critically re-examine the necessity of AED polytherapy in all patients, but especially the elderly, the institutionalized, children, and women, since they are especially vulnerable to undesirable combinations of AED and non-AED polytherapy and subsequent adverse effects posing significant risks [13,57,59]. Women of child bearing potential treated with polytherapy have an increased risk of giving birth to children with major congenital malformations if they become pregnant [13,59]. The effects of polytherapy on the developmental outcome of children born to mothers with epilepsy is unknown. Polytherapy has been associated with decreased patient compliance, reduced quality of life, and increased costs. Recently, polytherapy was also demonstrated to be a predictive factor for reduced bone mineral density in patients with epilepsy [23]. Maternal AED polytherapy has also been correlated with a heightened risk for cognitive and motor developmental delays in infants exposed in utero [76]. Therefore, reserving polytherapy for patients who have no other alternative is reasonable.

When reduction of polytherapy has been unsuccessful due to increased breakthrough seizures, another means of enabling polytherapy reduction is to reconsider non-pharmacologic approaches to augment management of the patient’s epilepsy. Additional diagnostic testing to explore candidacy for epilepsy surgery or VNS therapy should be strongly considered (as per Fig.**1**), since these treatments may afford patients a greater chance of reducing or eliminating polytherapy. Several retrospective studies comparing pre-operative to post-operative AED regimens following successful anterior temporal lobectomy consistently report successful elimination of polytherapy in the majority of patients, [44,65, 71,80] and a prospective randomized trial of carbamazepine monotherapy versus preoperative polytherapy demonstrated equivalent efficacy and reduced adverse effects in the monotherapy treatment group [32]. With adjunctive VNS therapy, many patients have been able to decrease total drug load by reducing the total number of AEDs used or decreased doses of individual AEDs without seizure exacerbation [38].

## CONCLUSION

Monotherapy is usually preferred over polytherapy whenever possible in epilepsy care. However, a substantial number of patients with intractable epilepsy may respond to AED polytherapy. Appropriate uses of more than one AED include transitional polytherapy during conversion to a new monotherapy, and chronic maintenance polytherapy in refractory patients. When a patient becomes seizure-free while receiving polytherapy, it may be possible to taper and gradually discontinue the baseline AED which has been previously ineffective or poorly tolerated. Randomized trials investigating rational polytherapy are needed to assess which combinations are most efficacious. AED treatment should be adjusted based on PDD/DDD ratios less than 2 to maximize tolerability; patient and drug specific characteristics should be considered to minimize drug-drug and drug-disease interactions and limit the incidence of adverse effects. Intractable epilepsy patients should be continuously reassessed; polytherapy should be maintained only when improved efficacy outweighs adverse effects. Eliminating unnecessary polytherapy benefits many patients by reducing adverse effects, drug interactions, and cost.

## Figures and Tables

**Table 1:** Proposed Pharmacological Targets of AEDs

Drug	Sodium Channels	Calcium Channels/Currents	GABA Receptors	GABA Synapse	Glutamate Receptors	Other
***Older AEDs***						
Benzodiazepines			+++			Abuse potential may limit use
Carbamazepine	+++	+	+			Modulates brain adenosine
Phenobarbital/primidone			+++			Abuse potential may limit use
Ethosuximide		+++ (Modulates T-type Currents)				Inhibits NADPH-linked aldehyde reductase (necessary for gamma-hydroxybutyrate (GHB) synthesis; GHB can induce absences)
Phenytoin	+++					
Valproate	+++	+ (Modulates T-type Currents)		+		
						
***Newer AEDs***						
Felbamate	++	++		++	++	Idiosyncratic Toxicity limits use
Gabapentin	+	++		+		
Lacosamide						Binds CRMP-2 receptor
Lamotrigine	+++	+				
Levetiracetam						Modulates presynaptic neurotransmitter release by SV2A receptor binding.
Oxcarbazepine	+++	+				
Pregabalin		++				
Rufinamide	++					+?
Tiagabine				+++		
Topiramate	++	++	++	+	++	+weak carbonic anhydrase inhibtion
Zoisamide	+++	++ (Modulates T-type Currents)				++facilitates catecholaminergic and dopaminergic neurotransmission; +weak carbonic anhydrase inhibition;

+++ Primary target;

++ Probable target;

+ Possible target.

Modified from: Kwan P, Brodie MJ.  Combination therapy in epilepsy: when and what to use.  Drugs 2006;66 (14): 1817-1829.

**Table 2:** Examples of Desirable and Undesirable AED Combinations

Combination	Desirability	Rationale
***Phenytoin or Carbamazepine, plus***		
Lamotrigine	–	EIAEDs decrease LTG, heightened pharmacodynamic neurotoxic adverse effects
Oxcarbazepine	–	EIAEDs decrease OXC, OXC inhibits CYP 2C19 and may increase PHT concentrations; increased neurotoxic adverse effects
Topiramate	–	EIAEDs decrease TPM, TPM inhibits CYP 2C19 and may increase PHT concentrations; increased neurotoxic adverse effects
Levetiracetam	+	Possible synergism with CBZ
***Lamotrigine or Oxcarbazepine, plus***		
Gabapentin	++	Potential synergism
Pregabalin	++	Potential synergism
Levetiracetam	++	Potential synergism
Topiramate	+	Potential synergism
Zonisamide	+/–	Possible additive effects, similar principle MOA
***Valproate or divalproex, plus***		
Phenytoin	–	Increased free PHT fraction and neurotoxic adverse effects; however, retrospective case series suggest synergism
Carbamazepine	–	Valproate increases CBZ-epoxide, similar principle MOA
Lamotrigine	+/–	Controlled clinical trial evidence for efficacy; however, increased risk of rash
Topiramate	+/–	Hypothetically may offset weight gain adverse effects; however, increase in neurotoxic adverse effects
Levetiracetam	++	Possible synergism
Zonisamide	+	Possible synergism

+ Potentially desirable combination;

++ Desirable combination.

– Potentially undesirable combination;

– – Undesirable combination.

+/– Data are conflicting.

**Table 3:** Common Representative AED Interactions in Clinical Practice

AED	Mediates CYP Induction	Mediates CYP Inhibition	Metabolism Inducible by CYP Enzyme	Metabolism Inhibited at CYP Enzyme	Protein Binding	Other
***Older AEDs***						
Carbamazepine	+++	++	++ (+Autoinduction)	++	++	-
Ethosuximide	-	-	++	++	-	-
Phenobarbital	++	-	+	+	-	+ (Antacids decrease absorption)
Phenytoin	+++	-	+	++	+++	+ (Antacids decrease absorption)
Primidone	**++**	**+**	**+**	**+**	**-**	**-**
Valproate	-	++	-	-	+++	+ (Drugs impacting glucuronidation may impact valproate concentrations; Food slows absorption)
***Newer AEDs***						
Felbamate	++	+	+++	-	-	+ (Inhibits beta oxidation (increasing valproate concentrations)
Gabapentin	-	-	-	-	-	saturable absorption at doses > 1800 mg/day
Lacosamide	-	-	-	-	-	-
Lamotrigine	+/-	-	+++	-	-	+++ (drugs impacting glucuronidation such as valproate affect lamotrigine concentrations)
Levetiracetam	-	-	-	-	-	-
Oxcarbazepine	+ (at doses above 1800 mg/day, ++)	++ (at high phenytoin concentrations)	++	-	-	-
Pregabalin	-	-	-	-	-	-
Rufinamide	+	-	+	-	-	+++ (valproate increases rufinamide concentrations by up to 70%)
Tiagabine	-	-	+++	-	+	-
Topiramate	+ (>200 mg, +++)	++ (at high phenytoin concentrations)	+++	-	-	-
Zonisamide	-	-	+++	+	-	-

Key:

+++ = principle mechanism for drug interactions with this drug;

++ = secondary mechanism of drug interactions with this drug;

+ = possible but less common mechanism of interaction with this drug.

**Table 4:** World Health Organization (WHO) Defined Daily Doses (DDD) Index Useful for Drug Load Calculations for the AEDs

AED	DDD/mg	DDD/g	ATC Code
***Older AEDs***			
Carbamazepine	1000	1.0	N03AF01
Clonazepam	8	0.008	N03AE01
Ethosuximide	1250	1.25	N03AD01
Phenobarbital	100	0.1	N03AA02
Phenytoin	300	0.3	N03AB02
Primidone	1250	1.25	N03AA03
***Newer AEDs***			
Felbamate	2400	2.4	N03AX10
Gabapentin	1800	1.8	N03AX12
Lacosamide	n.a.	n.a.	N03AX18
Lamotrigine	300	0.3	N03AX09
Levetiracetam	1500	1.5	N03AX14
Oxcarbazepine	1000	1.0	N03AF02
Pregabalin	300	0.3	N03AX16
Rufinamide	1400	1.4	N03AF03
Tiagabine	30	0.03	N03AG06
Topiramate	300	0.3	N03AX11
Valproate	1500	1.5	N03AG01
Vigabatrin	2000	2.0	N03AG04
Zonisamide	200	0.2	N03AX15

ATC Code = Anatomical Therapeutic Chemical Classification.

n.a. = not currently available.

As viewed on 4/28/09 on the World Wide Web at:  http://www.whocc.no/atcddd/
